# Pharmaceutical compounding of orphan active ingredients in Belgium: how community and hospital pharmacists can address the needs of patients with rare diseases

**DOI:** 10.1186/s13023-019-1154-x

**Published:** 2019-08-01

**Authors:** V. Vanhoorne, E. Peeters, I. Van Tongelen, K. Boussery, E. Wynendaele, B. De Spiegeleer, J. P. Remon, C. Vervaet

**Affiliations:** 1 Laboratory of Pharmaceutical Technology, Ghent University, Ottergemsesteenweg 460, 9000 Ghent, Belgium; 2 Pharmaceutical Care Unit, Ghent University, Ottergemsesteenweg 460, 9000 Ghent, Belgium; 3 Laboratory of Drug Quality and Registration, Ghent University, Ottergemsesteenweg 460, 9000 Ghent, Belgium

**Keywords:** Rare diseases, Orphan active ingredients, Pharmaceutical compounding

## Abstract

**Background:**

Pharmaceutical compounding of orphan active ingredients can offer cost-effective treatment to patients when no other drug product is available for a rare disease or during periods of drug product shortages. Additionally, it allows customized therapy for patients with rare diseases. However, standardized compounding formulas and procedures, and monographs are required to ensure the patients’ safety.

**Results:**

Standardized formulas and compounding procedures were developed for seven orphan active ingredients (L-arginine, sodium benzoate, sodium phenylbutyrate, L-carnitine, chenodesoxycholic acid, primaquine phosphate, pyridoxal phosphate) and one non-orphan molecule (sodium perchlorate) regularly compounded by hospital pharmacists for extemporaneous use. The stability of these formulations was evaluated over 3 months at refrigerated (5 °C) and standard storage conditions (25 °C/60%RH) using HPLC-based assays and a suitable shelf life was assigned to the formulations. Additionally, suitable analytical methods for quality control of formulations of pyridoxal phosphate and sodium perchlorate were developed as monographs for these components were not available in the European Pharmacopeia or United States Pharmacopeia.

**Conclusions:**

Availability of compounding formulas and protocols, as well as stability information, for orphan active ingredients can improve patients’ access to treatment for rare diseases. Such data were collected for seven orphan active ingredients to treat patients with rare diseases when no other treatment is available. More efforts are needed to develop standardized formulas and compounding procedures for additional orphan active ingredients whose clinical efficacy is well-known but which are not available as products with a marketing authorization. Additionally, a legal framework at EU level is required to enable the full potential of pharmaceutical compounding for orphan active ingredients.

## Background

In the European Union (EU), rare diseases are defined as life-threatening or chronically debilitating conditions that affect no more than five in 10 000 patients [1]. It is estimated that 5000 to 8000 rare diseases exist worldwide, while approximately 250 new diseases are described annually [1–3]. Although their prevalence is low, rare diseases affect 27 to 36 million people in the EU [1, 2]. Orphan medicinal products (OMPs) are defined by the EU as drug products intended for a condition for which there exists no authorized satisfactory method of diagnosis, prevention or treatment or, if so, that it will be of significant benefit to those affected by the condition [1]. The development of and patient access to OMPs is therefore an important challenge for public health policymakers. However, the challenges associated with OMPs are manifold and are mainly related to lack of research and development of OMPs and patient access to OMPs.

The low number of patients with a particular rare disease can limit OMP development due to the risk for pharmaceutical companies to achieve sufficient return on their investment and the difficulty in recruiting sufficient numbers of patients to prove statistically significant effects in clinical trials [3]. The economical aspect was addressed by the Orphan Drug Act in the United States in 1983 and the adoption of the European Commission Regulation Number 141/2000 and 847/2000 in 2000, offering economic and regulatory incentives [3]. In the EU, these include protocol assistance, 10-year market exclusivity and fee waivers for regulatory procedures for designated products for companies investing in the research and development of OMPs [3]. Additionally, the US Food and Drug Administration (FDA) and the European Medicine Agency (EMA) have enabled a common EMA-FDA application for orphan designation. Next to OMP development, patient access is essential but is often limited by high costs and lack of reimbursement [4]. As pricing and reimbursement of OMPs are EU member state responsibilities, patient access varies within the EU. The EU Commission therefore set up a working party to exchange knowledge between member states on scientific assessment of the clinical added value of orphan medicines that can facilitate national pricing and reimbursement decisions [5].

An increasing number of OMP designations and authorizations have been reported in the EU since 2000 [3, 6]. From 2000 to 2017 the EMA reported 1952 active OMP designations (i.e. designation of the status of OMP to a drug product) while 142 products received market authorization (i.e. a license to market a drug product) [6]. This is a success considering that there was almost no development of OMPs in the EU prior to 2000 [3, 6]. However, this also signifies that for less than 2% of rare diseases, authorized medicinal products are available in the EU and that continued efforts from the EU and its member states are required [2]. Therefore, the EU Commission and Council obligated their member states to develop dedicated national strategies to pursue a comprehensive and integrated approach to the delivery of health and social care for rare disease patients [1, 5].

In Belgium, the Fund for Rare Diseases and Orphan Drugs drafted a report including 42 recommendations and proposals for a national plan for rare diseases [7]. These recommendations are related to (1) expertise and multi-disciplinarity, (2) collaboration and networking, (3) knowledge, information and awareness, (4) equity in access, (5) governance and sustainability [7]. A measure to improve equity in access comprises creation of a legal framework to use orphan active ingredients (OAIs) in compounded preparations. Simultaneously, compounding of OAIs could offer opportunities to cut costs in the health care budget. Based on the latest available figures, the Belgian social security services reimbursed an increasing number of OMPs from 2009 to 2016, with expenditures rising from around 150 million euro in 2009 up to more than 350 million euro in 2016, which accounts for a rise from around 4% to up to more than 8% of the budget spent on medicine reimbursement [8]. Begin 2018, 149 OMPs had a marketing authorization and 61% of these were reimbursed [8]. Some OMPs were not reimbursed because alternatives exist that are more cost-effective or due to budgetary restrictions, while for others no reimbursement was applied for or the application procedure is still ongoing [8].

Some well-known OAIs for treatment of orphan diseases are not available to patients as products with a marketing authorization. Consequently, off-label use is widespread for treatment of orphan diseases [9]. However, compounding of OAIs could address these patients’ needs. Additionally, compounding of OAIs can address the needs of pediatric or geriatric patients, offering dosing flexibility, and of patients with hypersensitivity, intolerances and allergies to specific excipients of authorized drug products. However, three main obstacles must be overcome to use OAIs in compounded preparations. Firstly, compounding of OAIs should be performed by pharmacists applying standardized compounding procedures and formulas, with knowledge of the stability, in order to assure patient safety [10]. Secondly, monographs for pharmaceutical analysis of OAIs are often not available, hence suitable analytical methods must be developed for quality control of these compounds, reflected in their certificates of analysis, as well as for the compounded formulations. Thirdly, there is a lack of EU-uniform legal framework to use OAIs in compounded preparations. Despite these hurdles, some OAIs are currently used in enteral and parenteral preparations compounded in community and hospital pharmacists in Belgium and other countries when no other treatment is available for patients with rare diseases [11–14]. Nevertheless, to ensure the patients’ safety and to improve the equity in access, efforts are needed to develop standardized compounding procedures and formulas, to develop quality monographs (if none are available) and to create a legal framework.

The Laboratory of Drug Quality and Registration and the Laboratory of Pharmaceutical Technology at Ghent University took the initiative to develop formulas and standard compounding procedures and to develop suitable analytical methods for quality control of the compounded formulations in order to promote pharmaceutical compounding of OAIs by hospital and community pharmacists. These formulas and procedures are outlined in the current paper.

## Methods

### Materials

An overview of the OAIs, excipients and packaging used in the formulations is given in Table 1.

**Table 1:** Overview of the OAIs, excipients and packaging used in the compounded formulations

OAIs	Supplier
L-arginine	Fagron, Waregem, Belgium
L-carnitine	Fagron, Waregem, Belgium
Chenodesoxycholic acid	BOC Sciences Creative Dynamics, Shirley, United States
Primaquine phosphate	BOC Sciences Creative Dynamics, Shirley, United States
Pyridoxal phosphate	Inresa, Bartenheim, France
Sodium benzoate	Fagron, Waregem, Belgium
Sodium perchlorate^a^	Merck, Darmstadt, Germany
Sodium phenylbutyrate	Fyrklovern Scandinavia AB, Mönsteras, Sweden
Excipients	Supplier
*Aurantii amari epicarp et mesocarp tinctura*	Conforma, Destelbergen, Belgium
*Sirupus simplex*	Conforma, Destelbergen, Belgium
Methyl paraben	Fagron, Waregem, Belgium
Propyl paraben	Fagron, Waregem, Belgium
Mannitol	Fagron, Waregem, Belgium
Colloidal silicon dioxide	Fagron, Waregem, Belgium
Packaging	Supplier
Hard gelatine capsules	Capsugel, Bornem, Belgium
Glass bottles 100 ml	Aca Pharma, Nazareth, Belgium
Plastic opaque cups	Aca Pharma, Nazareth, Belgium

^a^Sodium perchlorate is not an OAI but was included in the study as an unlicensed molecule regularly compounded by hospital pharmacists for extemporaneous use

### Methods

#### Selection of orphan active ingredients

A list of OAIs for use in galenic preparations and showing an unmet medical need was composed by an expert panel, consisting of 20 hospital pharmacists, and approved by the Belgian National Formulary commission. These OAIs were scored based on (A) the prevalence of the corresponding rare disease, (B) severity of the disease, (C) degree of available clinical and therapeutical evidence, (D) price of the OAI and (E) ease of compounding. Scores from 1 (for questionable/low) to 3 (for evident/high) were assigned to these criteria. Next, a weighing factor was assigned to the criteria depending on their importance (Table 2). Finally, the total score was calculated by summing the scores of the individual criteria multiplied with the corresponding weighing factor (Table 2). All active ingredients included in current study obtained orphan designation by EMA and/or FDA [15, 16].

**Table 2:** Priority list of the OAIs

	A (2)	B (4)	C (3)	D (2)	E (1)	total
Amifampridine	6	12	9	6	3	36
Sodium phenylbutyrate^a^	6	12	9	2	3	32
Pyridoxal phosphate^a^	6	8	9	6	3	32
Chenodesoxycholic acid^a^	2	12	9	6	3	30
Primaquine phosphate^a^	2	12	9	6	3	30
Fenfluramine	2	12	6	6	3	29
Sodium hydroxybutyrate	2	12	3	6	3	26
Difencyprone	6	4	6	6	3	25
Bimyconase	2	8	6	2	1	19

Priority list of the OAIs based on five criteria with (A) the prevalence of the corresponding rare disease, (B) severity of the disease, (C) degree of available evidence, (D) price of the orphan active ingredient and (E) the ease of compounding. The weighing factor (which was multiplied with the score of the individual criterium) indicating the criteriums importance is mentioned between brackets. ^a^OAIs included in current study

#### Development of compounding formulations

The OAIs were compounded as liquid (solutions) or solid (capsules) formulations intended for oral administration. The choice for a specific dosage form depended on the OAI dose, dosing frequency, patient population, solubility and sensitivity to air, heat and light. The OAI concentration in the liquid formulations and the OAI content per capsule was based on scientific literature and the expert panel’s advice (Table 3).

**Table 3:** Overview of the OAIs included in current study

	Orphan disease	Prevalence (/100.000) or number of published cases [15]	Recommended dose	Reference	Developed dosage form with OAI concentration (solutions) or OAI mass per dosage unit (capsules)
L-arginine, sodium benzoate, sodium phenylbutyrate	Hyperammonemia: carbamoyl Phosphate synthase deficiency	0.31P	L-arginine: < 20 kg: 100–200 mg/kg/day; > 20 kg: 2.5–6 g/m^2^/day (max. 6 g/day) Sodium benzoate: up to 250 mg/kg/day (max. 12 g/day) Sodium phenylbutyrate: < 20 kg: ≤ 250 mg/kg/day; > 20 kg: 5 g/m^2^/day (maximum: 12 g/day)	[17]	L-arginine: 10 g/100 ml solution Sodium benzoate: 10 g/100 ml solution Sodium phenylbutyrate: 20 g/100 ml solution
	Hyperammonemia: ornithine transcarbamylase deficiency	1.4P*			
	Hyperammonemia: citrillinaemia or argininosuccinate synthase deficiencyHyperammonemia: argininosuccinic aciduria or argininosuccinate lyase deficiency	2.4P*1.0P*	L-arginine: < 20 kg: 100–300 mg/kg/day; > 20 kg: 2.5–6 g/m^2^/day (max. 6 g/day) Sodium benzoate: up to 250 mg/kg/day (max. 12 g/day)Sodium phenylbutyrate: < 20 kg: ≤ 250 mg/kg/day; > 20 kg: 5 g/m^2^/day (maximum: 12 g/day)	[17]	
	Hyperammonemia due to N-acetylglutamate synthase deficiency	12^a^	L-arginine: < 20 kg: 100–200 mg/kg/day; > 20 kg: 2.5–6 g/m^2^/day (max. 6 g/day) Sodium benzoate: up to 250 mg/kg/day (max. 12 g/day)	[17]	
L-carnitine	Carnitine palmitoyl transferase1A deficiency	50 cases	N.A.		20 g/100 ml solution
	Carnitine-acylcarnitine translocase deficiency	60 cases	N.A.		
	Medium-Chain Acyl-Coenzyme A Dehydrogenase Deficiency	3.2 BP^a^	100 mg/kg/day	[18]	
	Systemic primary carnitine deficiency	6.85 P	100–400 mg/kg/day	[19]	
Sodium Benzoate	Non-ketotic hyperglycemia Glycine encephalopathy	0.17 P*	250–750 mg/kg/day	[20]	10 g/100 ml solution
Chenodesoxycholic acid	Cerebrotendinous xanthomatosis	< 5 P	750 mg/day	[21, 22]	250 mg capsules
Primaquine phosphate	Malaria	3.0 P*	500 μg/kg and 250 μg/kg daily for 14 days	[23–26]	30 mg capsules
	*Pneumocystis carinii* pneumonia	N.A.	15 mg/day		
Pyridoxal phosphate	Pyridoxamine 5′-oxidase deficiency	0.2 P*	30 – 50 mg/kg/day	[27, 28]	10 mg capsules
Sodium perchlorate^a^	Prophylactic thyroid protection during radiological examination using iodine contrast agents	N.A.	1 g/day	[29]	50 mg/ml solution

Overview of the OAIs included in current study, the corresponding orphan diseases, overall prevalence worldwide (P) or in Europe (P*), birth prevalence worldwide (BP) or number of published cases, recommended dose and dose of compounded formulation (N.A.: data not available). ^a^Sodium perchlorate is not an OAI but was included in the study as an unlicensed molecule regularly compounded by hospital pharmacists for extemporaneous use

The preparation of the formulations was standardized following the best practices described in the Belgian National Formulary. Capsules were prepared using a mannitol/colloidal silicon dioxide mixture (99.5/0.5 w/w%) as a non-hygroscopic, non-reducing filler. Capsule size number 2 or larger was used. Capsules were packaged in plastic opaque cups of 100 ml. Solutions were compounded by dissolving the AOI in a glass flask in 50 ml demineralized water containing 0.02% propylparahydroxybenzoate (PBA) and 0.08% methylparahydroxybenzoate (MBA). Next, at least 85 g *sirupus simplex* (consisting of 65.00 w/w% sucrose, 0.02% propylparahydroxybenzoate and 0.08% methylparahydroxybenzoate in deionized water) and 3 g *aurantii amari epicarp et mesocarp* tincture were added. Finally, the preparation was diluted with *sirupus simplex* up to a total volume of 100 ml. The solutions were filled into transparent glass flasks, except for the L-arginine solution which was filled into a brown glass flask to avoid degradation through light exposure [30].

#### Stability testing

Identification, assay, related substances (i.e. degradation products), mass uniformity (for capsules) and pH (for solutions) tests were conducted on the preparations immediately after preparation (T0), after 3 months storage at 5 °C (T3_5°C), after 1 (T1_25°C/60%RH) and 3 (T3_25°C/60%RH) months storage at 25 °C and 60% relative humidity (RH) in constant climate chambers (Pharma 500 l, Weiss Technik, Liedekerke, Belgium). For chenodesoxycholic acid capsules, no data was collected after 3 months but after 4 months storage. Maximal storage duration of 3 or 4 months was considered sufficient, as the compounded formulations are delivered to the patient immediately after preparation. Due to detection of degradation products in the arginine solutions, assay determination of MBA and PBA in the arginine solutions was performed after 1 and 2 months storage under two conditions: (1) in the fridge (5 °C) and (2) at 25 °C and 60% RH (no data was collected after 3 months storage as clear degradation of the parabens was already detected after 2 months). Additionally, all samples were visually inspected at all timepoints for color change and evaluated for odor.

All identification, assay and related substances (i.e. degradation products) tests, except for chenodesoxycholic acid and sodium perchlorate, were performed using in-house developed and validated high-performance liquid chromatography (HPLC) methods with ultraviolet (UV) spectroscopy detection. Identification, assay and related substances tests, of sodium perchlorate were performed using an in-house developed and validated UPLC method with mass spectrometry detection. The details of these analytical methods are given in Table 4. Identification and determination of the related substances, impurities and degradation products of chenodesoxycholic acid was performed by thin layer chromatography as described in its European Pharmacopeia (Ph. Eur.) monograph whereas assay of chenodesoxycholic acid itself was performed by acid-base titration using an aqueous sodium hydroxide solution [31].

**Table 4:** Overview of the applied analytical methods

	Column HPLC	Mobile phase	Flow rate (ml/min)	UV detection wavelength (nm)	Column oven temperature (°C)	Injection volume (μl)
L-arginine	Nucleodur NH2-RP, 5 μm, 4.6 mm × 150 mm	Isocratic: phosphate buffer pH 7.0/acetonitrile 47/53	0.8	210	30	20
L-carnitine	Nucleodur NH2-RP, 5 μm, 4.6 mm × 150 mm	Isocratic: phosphate buffer pH 4.7/acetonitrile 40/60	0.8	205	30	20
Primaquine phosphate	Symmetry C8, 3.5 μm, 3 mm × 150 mm	Isocratic: acetonitrile/tetrahydrofuran/trifluoro acetic acid/water 9/1/0.1/90	0.75	265	30	10
Pyridoxal phosphate	Prevail C18, 5 μm, 4.6 mm × 250 mm	Isocratic: 25 mM KH_2_PO_4_ pH 3/acetonitrile 97/3	1	212	25	20
Sodium benzoate	Prevail Organic acid, 5 μm, 4.6 mm × 250 mm	Isocratic: Water/acetonitrile 75/25 + 0.1% (m/V) formic acid	1.0	225	25	20
Sodium perchlorate	Primesep D column, 5 μm, 3.2 mm × 150 mm equipped with suitable guard column	Gradient with A: 10/90 acetonitrile/water (V/V) + 30 mM ammonium formate, B: 40/60 acetonitrile/water + 80 mM ammonium formate	0.6	-^a^	30	1
Sodium phenylbutyrate	C18, end-capped, base-deactivated, 5 μm	Isocratic: glacial acetic acid/methanol/water 1/49/50	1.1	245	35	20
MBA, PBA	C18, 5 μm, 4.6 mm × 150 mm	Gradient with A: 95/5 0.62% (m/V) KH_2_PO_4_ in water/methanol (V/V), B: 5/95 0.62% (m/V) KH_2_PO_4_ in water/methanol (V/V)	1.2	245	25	10

Overview of the column type, mobile phase, flow rate, UV detection wavelength, column oven temperature and injection volume applied in the HPLC methods. A gradient was used for analysis the sodium perchlorate formulation (0 min: 100% A, 0% B; 12 min: 0% A, 100% B, 12.5 min: 100% A, 0% B) and for assay of MBA, PBA in the L-arginine formulation (0 min: 90% A, 10% B; 17 min: 36% A, 64% B; 22 min: 90% A, 10% B). ^a^Detection of sodium perchlorate was performed by mass spectrometry

An OAI was identified when its retention time was within ±0.5 min of the retention time of the OAI peak in a reference standard (the active ingredient used in the formulation) or when the principal spot on the thin layer chromatogram of the test solution was similar in position, color and size to the principal spot in the chromatogram obtained with the reference solution. For assay determination via HPLC, two reference solutions and two sample solutions were prepared independently and analyzed in duplicate. Standard curves of each component were constructed via linear regression of the peak area to the concentration and used to calculate the percentage label claim. The formulations complied to the assay test when the assay of the solutions and capsules was within a 90–110% label claim and 85–115% label claim interval, respectively. A reporting threshold of 0.1% label claim was applied for related substances (i.e. degradation products) according to the Ph. Eur. monograph on substances for pharmaceutical use, unless other limits were included in the specific Ph Eur. monograph of a OAI [32]. The mass uniformity of the capsules was evaluated at release and during stability testing according to the monograph for uniformity of mass of single dose preparations of the Ph. Eur. [33].

## Results

### Selection of orphan active ingredients

A priority list based on the scores assigned to the unlicensed OAIs defined by an expert panel of the Belgian National Formulary is given in Table 2. It was decided to develop standard compounding procedures for the OAIs with the five highest scores. Although amifampridine scored highest, no compounding procedure was developed as this is already available in the German National Formulary (Deutsche Artsenei Codex). Standardized formulas and compounding procedures were developed for sodium phenylbutyrate, pyridoxal phosphate, chenodesoxycholic acid and primaquine phosphate. Additionally, standardized formulas and compounding procedures were developed for three licensed OAIs which are frequently compounded in Belgian hospital pharmacies but whose stability was not investigated up to now: L-arginine, L-carnitine and sodium benzoate. Sodium perchlorate, which is not an OAI but an unlicensed molecule, was also included in the study based on the input of the expert panel as sodium perchlorate is frequently compounded by hospital pharmacists for extemporaneous use prior and after radiological examination of the thyroid, although stability data was lacking up to now.

An overview of the compounded OAIs, their recommended dose and the corresponding developed formulation is presented in Table 3. Additionally, the rare diseases and their prevalence for the corresponding OAIs are included in Table 3.

Next to the standardized formulas and compounding procedures, suitable analytical methods were developed for quality control of pyridoxal phosphate and sodium perchlorate formulations as no monographs for these components were available in the Ph. Eur. or USP. Details of these tests are included in Table 4.

### Stability testing

**Fig. 1:**
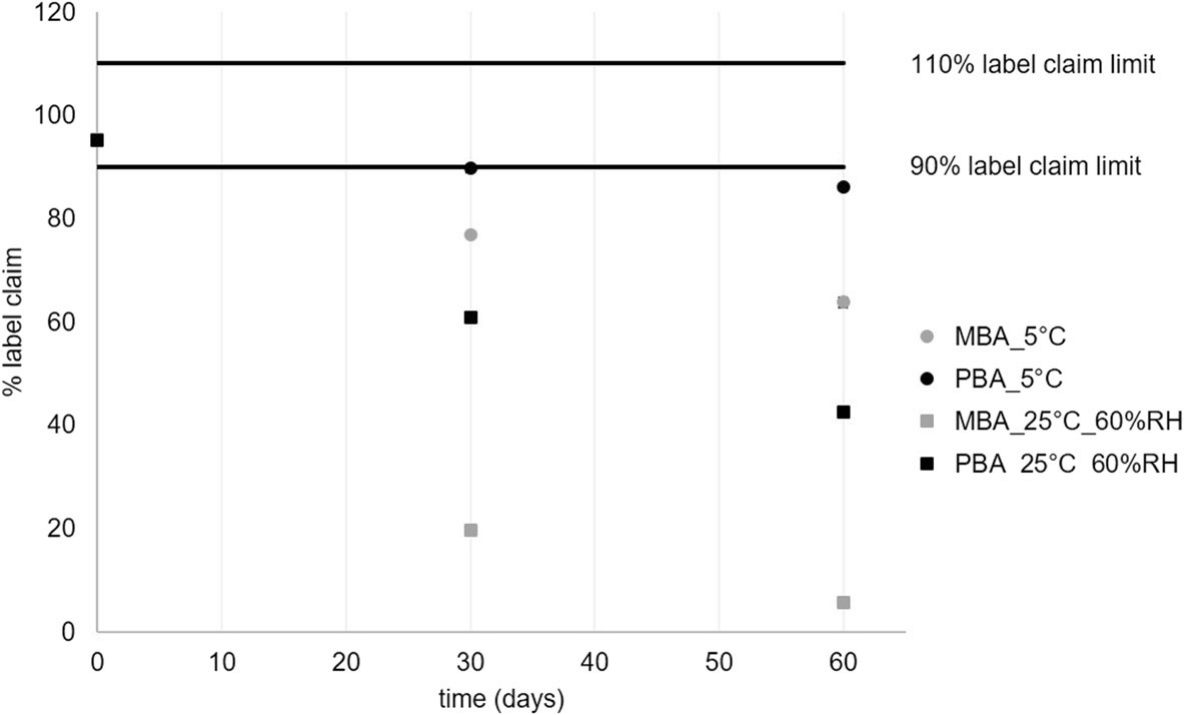
Label claim% of MBA and PBA in the L-arginine solution in function of time. Label claim % of MBA (grey) and PBA (black) included in the L-arginine solution after storage at 5 °C (●) or 25 °C and 60%RH (■)

The suitability of the developed formulas and compounding procedures was evaluated via stability testing to investigate how long the extemporaneously produced preparations can be stored and which storage conditions should be applied to ensure their activity. All formulations complied with the mass uniformity test, identification and related substances tests at release. Additionally, all formulations complied with the mass uniformity and identification tests during stability testing. An overview of the assay results at the investigated time points is presented in Table 5. The L-carnitine, sodium benzoate and sodium phenylbutyrate solutions and chenodesoxycholic acid, pyridoxal phosphate and primaquine phosphate capsules were stable over 3 months under all storage conditions. All formulations, except the L-carnitine and L-arginine solutions, complied to the specifications for related substances (i.e. degradation products). A degradation peak (RRT 0.89) exceeding the reporting threshold (0.10% label claim) was detected in the L-carnitine solutions after 1 (0.24% label claim) and 2 months (0.17% label claim) only (and not after 3 months). However, as the assay of L-carnitine was stable over 3 months and as the detected degradation peak was a broad HPLC-peak, which is atypical for a well-identified degradant, it was assumed that the observed degradation peak was due to degradation of the aroma, *aurantii amari epicarp et mesocarp*, which is a complex mixture [34, 35]. Therefore, a shelf life of 3 months was assigned to the L-carnitine solutions. L-arginine was stable over the tested time period, but degradation of PBA and MBA in the L-arginine solution was observed after 1 month storage at both refrigerated and 25 °C/60% RH conditions in a brown glass flask. Paraben degradation was attributed to hydrolysis in alkaline environment, as paraben hydrolysis is commonly observed at pH values exceeding 7.0, and as the pH of the L-arginine solutions was 10.5. An overview of the paraben decrease over time in function of the storage conditions is given in Fig. 1. Faster degradation was observed for MBA compared to PBA which was attributed to the stronger resistance to hydrolysis observed with an increase in alkyl chain length of parabens [36]. Assuming linear degradation kinetics, based on the collected data points, the arginine solution could be stored for maximum 9 days under refrigerated conditions. Therefore a shelf life of 1 week under refrigerated conditions was recommended (Table 5).

**Table 5:** Overview of the shelf life and assay stability results

Assay	T0	T3_5°C	T1_25°C/60%RH	T3_25°C/60%RH	Shelf life
L-arginine solution	98.7; 98.8	100.3; 99.9^a^	100.8; 99.9^a^	98.5; 97.4^a^	1 week refrigerated (5 °C)
L-carnitine solution	98.6; 100.8	102.7; 102.8	103.5; 102.9	99.3; 99.9	3 months
Chenodesoxycholic acid capsules	96.9; 96.3; 96.6	97.6; 97.6; 97.6	98.9; 99.4; 99.3	101.2; 100.4^b^	3 months
Primaquine phosphate capsules	105.7; 101.8	98.4; 99.7	95.3; 96.7	95.3; 99.7	3 months
Pyridoxal phosphate capsules	104.9; 104.6	105.5; 101.8	100.3; 100.3	101.2; 100.2	3 months
Sodium benzoate solution	99.0; 99.5	101.8; 101.6	100.2; 100.1	100.6; 97.2	3 months
Sodium perchlorate solution	90.4; 96.18	92.1; 90.1	90.6; 93.2	91.7; 89.12	3 months
Sodium phenylbutyrate solution	99.7; 99.5	99.4; 101.5	100.0; 100.9	96.7; 99.7	3 months

Overview of the shelf life and assay stability results (*n* = 2 or *n* = 3) immediately after production (T0), after 3 months refrigerated storage (T3_5°C), after 1 (T1_25°C/60%RH) and 3 (T3_25°C/60%RH) months storage at 25 °C and 60% RH. Formulations complied to the assay test when the assay of the solutions and capsules was within a 90–110% label claim and 85–115% label claim interval, respectively. ^a^Degradation products of PBA and MBA were detected, ^b^Stability data was not collected after 3 months but after 4 months

## Discussion

The developed formulas and compounding procedures for the selected OAIs can offer patient access to treatment of rare diseases when no commercial orphan medicinal product (OMP) is on the market, provided a legal framework is created. Additionally, similar work should be performed for other OAIs that are not commercially available to address the needs of patients with rare diseases. Several OAIs were suggested in the report of the Belgian Fund for Rare Diseases and Orphan Drugs or by the expert panel including d,I-3-sodiumhydroxybutyrate, amiloride, bi-myconase, I-citrulline, CoEnzyme Q10, diphencyprone, d-mannose, d-ribose, fenfluramine, glycine, hydroxobalamine, oxybutinine, squaric acid dibuty ester, potassium citrate, propranolol and sodium thiosulphate [7].

Next to increasing patient access, compounding of OAIs can also be an interesting way to cut costs in the portion of the health care budget dedicated to rare diseases. Although some OMPs are novel or complex new chemical entities developed at high research and development (R&D) costs, such high R&D costs are not applicable to others as either evidence of their efficacy has already been published in literature prior to market authorization, or because the drug product was already developed for another diseases, decreasing to some extend the costs in the quality and preclinical field [37, 38]. In this context, Simoens et al. compared the price of commercially manufactured OMPs to compounding costs in a community pharmacy for a selection of orphan drugs on the Belgian market in 2011. The price of commercially manufactured OMPs exceeded 2- to 148-fold the compounding costs for the studied OMP [38].

While standardized formulas and compounding procedures were developed for seven OAIs in current study, monographs and compounding procedures for other OAIs are included in the German National Formulary (e.g. cysteamine, polyhexamethylbiguanide, amifampridine), USP (e.g. mepacrine) or others. Dooms et al. listed national formularies and other reliable sources available worldwide to retrieve information on compounding of OAIs for rare diseases [10]. To realize all benefits of compounding OAIs, it is essential to create a legal framework for their use. Preferably, this should be organized at EU level by creation of an authority listing and certifying OAIs and issuing a formulary including standardized formulas and compounding procedures.

## Conclusions

Compounded preparations of OAIs can improve patients’ access to treatment for rare diseases. However, they should be prepared in accordance to standardized formulas and compounding procedures and the quality of the OAI should be demonstrated by a certificate of analysis according to a specific monograph. In current study, such standardized formulas and compounding procedures were developed for seven OAIs to treat patients with rare diseases when no other treatment is available. More efforts are needed to develop standardized formulas and compounding procedures for additional OAIs whose clinical efficacy is well-known but are not available yet to patients due to lack of interest from the pharmaceutical industry to apply for market authorization as OMP. Additionally, a legal framework at EU level is required to enable the potential of pharmaceutical compounding for OAIs.

## Data Availability

The datasets used and/or analysed during the current study are available from the corresponding author on reasonable request.

## Abbreviations

EMA: European Medicine Agency
EU: European Union
FDA: Food and Drug Administration
HPLC: High-performance liquid chromatography
MBA: Methylparahydroxybenzoate
OAI: Orphan Active Ingredient
OMP: Orphan Medicinal Product
PBA: Propylparahydroxybenzoate
Ph. Eur.: European Pharmacopeia
R&D: Research and development
USP: United States Pharmacopeia
UV: Ultraviolet
